# Autonomous Assessment of Delamination Using Scarce Raw Structural Vibration and Transfer Learning

**DOI:** 10.3390/s21186239

**Published:** 2021-09-17

**Authors:** Asif Khan, Salman Khalid, Izaz Raouf, Jung-Woo Sohn, Heung-Soo Kim

**Affiliations:** 1 Department of Mechanical, Robotics and Energy Engineering, Dongguk University Seoul, 30 Pildong-ro 1 Gil, Jung-gu, Seoul 04620, Korea; asif_dgu@dongguk.edu (A.K.); salmankhalid@dongguk.edu (S.K.); izazraouf@dongguk.edu (I.R.); 2 Department of Mechanical Design Engineering, Kumoh National Institute of Technology, Gumi 39177, Korea

**Keywords:** laminated composites, structural vibration, synchroextracting transform, scarce data, autonomous features

## Abstract

Deep learning has helped achieve breakthroughs in a variety of applications; however, the lack of data from faulty states hinders the development of effective and robust diagnostic strategies using deep learning models. This work introduces a transfer learning framework for the autonomous detection, isolation, and quantification of delamination in laminated composites based on scarce low-frequency structural vibration data. Limited response data from an electromechanically coupled simulation model and from experimental testing of laminated composite coupons were encoded into high-resolution time-frequency images using SynchroExtracting Transforms (SETs). The simulated and experimental data were processed through different layers of pretrained deep learning models based on AlexNet, GoogleNet, SqueezeNet, ResNet-18, and VGG-16 to extract low- and high-level autonomous features. The support vector machine (SVM) machine learning algorithm was employed to assess how the identified autonomous features were able to assist in the detection, isolation, and quantification of delamination in laminated composites. The results obtained using these autonomous features were also compared with those obtained using handcrafted statistical features. The obtained results are encouraging and provide a new direction that will allow us to progress in the autonomous damage assessment of laminated composites despite being limited to using raw scarce structural vibration data.

## 1. Introduction

The use of composite materials is growing continuously in a variety of industries (e.g., aerospace, automotive, wind energy) and remains at the forefront of contemporary research [1,2,3,4,5]. These materials have preferential properties of high specific stiffness, high specific strength, high corrosion resistance, being lightweight, and offering design flexibility. Despite their benefits, the orthotropic layered nature of laminated composites makes them prone to complex failure modes (matrix cracks, fiber breakage, delamination, etc.) during their manufacturing and in-service life [6]. Among the various defects, delamination is one of the most commonly occurring failure mode in laminated composites. The presence and propagation of delamination can result in up to a 60% loss in structural stiffness without any visible change to the surface of the material [7,8]. Owing to these severe consequences, developing a diagnostic strategy to continuously assess laminated composites for the presence of delamination is imperative. Commonly employed non-destructive evaluation methods (ultrasonic C-scan, shearography, computed tomography, etc., [9,10] are not suitable for continuous real time in-service damage assessments.

In recent years, AI-based machine learning and deep learning techniques have emerged as powerful tools that can be used for early damage detection in composite structures in various applications such as helping airplanes avoid catastrophic failures. Ai et al. [11] adopted a model based on random forest and deep learning methods to perform impact localization in aircraft composite structures using acoustic emission (AE) data. The analysis of the results showed that the random forest and deep learning model achieved better event localization performance than previous conventional artificial neural networks. Brien et al. [12] and his team used acoustic emission signals generated from healthy and faulty composite beams to test their proposed pattern recognition system that employs an artificial neural network classifier to detect and classify damage in composite beams. The proposed methodology was proven to be able to effectively classify damage into one of four different levels with 97% accuracy. Khan et al. [13] proposed a Convolutional Neural Network (CNN) approach to analyse the low-frequency structural vibration outputs generated by composite laminates that came from either healthy cases or from 12 delaminated cases. The output results showed the proposed model could classify cases as healthy or delaminated with 90.1% accuracy. Loutas et al. [14] developed an intelligent structural health monitoring system for composite structures used in aerospace applications that used multi-sensor data fusion in a feature level approach to classify thirteen different damage states. The results indicated that using SVM with a nonlinear kernel could detect faults with up to 99.3% accuracy. Further, various authors have applied machine and deep learning models in these references [15,16,17,18] for damage detection and classification of composite structures. Although deep learning models with several hidden layers are able to autonomously discover discriminative features from target images, a substantial amount of annotated data is required to optimize the network parameters in these systems. In engineering applications, acquiring sufficient data with faulty states is difficult due to the severe consequences of operating a system in the presence of any kind of fault.

From the literature survey above, it is clear AI-based models are able to detect and classify faults in composite structures with a high level of accuracy. In cases with small training datasets, the trained model will have poor generalization. The small scale of the data in these situations cannot represent the distribution to which they belong, and the training and testing datasets must have the same distribution. On the other hand, large-scale training data is expensive and challenging to collect in certain scenarios such as aerospace applications. To overcome this issue, transfer learning-based models have been introduced recently for intelligent fault detection. Gong et al. [19] proposed a novel deep transfer learning model for aeronautics composite materials (ACM) that combined deep learning with a sliding window approach. The proposed model used X-ray images of ACM but these samples were scarce. Experimental results showed that the proposed model could classify faults up to 96% classification accuracy. Outside of fault detection systems, Milad et al. [20] used a transfer learning approach with smart manufacturing processes of composite materials. The historical data generated from their autoclave curing process was successfully used to learn new cycle settings with limited data. Yang et al. [21] proposed a feature-based transfer neural network for bearing fault detection that used transferred diagnosis knowledge from laboratory bearings to detect faults in locomotive bearings. The results showed that the proposed model could successfully identify transferable features from the laboratory scale bearings to detect faults in locomotive bearings. Other literature on applying transfer learning to intelligent fault diagnosis can be found in the references [22,23,24,25].

The current work proposes aiding autonomous feature extraction from limited data using off-the-shelf pretrained deep learning models for the assessment of delamination in laminated composites. An electromechanically coupled finite element model was employed to study the effect of different sized delaminations based on a numerical framework. For experimental verification of the proposed approach, laminated composite samples were manufactured using a hot press machine and water cutting jet. Low-frequency structural vibration responses were obtained from simulations and from the experimental samples. The time series data was transformed into time-frequency images using the Synchroextracting Transform (SET) technique [26,27]. Pretrained deep learning models based on AlexNet [28], GoogleNet [29], SqueezeNet [30], ResNet-18 [31], and VGG-16 [32] were employed to extract low- and high-level features from the images based on the limited simulated and experimental data. A conventional machine learning algorithm, support vector machines (SVM), was used to assess the extracted autonomous features for their usefulness in the detection, isolation, and quantification of delamination in laminated composites. The classification performance using the autonomous features was also compared with the results from using handcrafted statistical features.

## 2. Proposed Methodology

**Figure 1:**
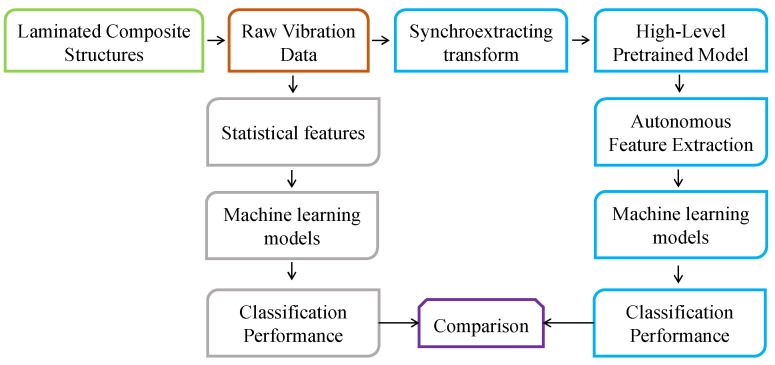
The general workflow of the proposed methodology.

This section describes the proposed methodology. The general workflow of the current work is shown in Figure 1.

First, structural vibration responses were obtained from the laminated composite plates in both pristine and delaminated states. The raw vibration signals were processed via the Synchroextracting Transform (SET) technique to produce high resolution time-frequency “images” that represent the signals in such a way that they can be used as inputs to deep learning-based diagnostic systems. High-level deep learning models were employed to autonomously extract discriminative feature from the limited data encoded in the time-frequency images. These autonomous features were processed using different machine learning models and the performance of each was evaluated in terms of training, validation, testing, and confusion matrices. To show the effectiveness of the proposed approach to diagnose laminated composites, handcrafted statistical features from the time and frequency domains were extracted from the raw vibration signals and processed with machine learning models. The results achieved using the handcrafted statistical features were also evaluated in terms of training, validation, testing, and confusion matrices so they could be compared to those achieved based on autonomous feature extraction. Each approach was evaluated based on input data from numerical simulations as well as real-world experiments on laminated composites with delaminations of various sizes. In general, high frequencies excitation and response signals (such as guided waves) are more suitable for detecting and isolating small size delamination defects. However, the delamination detection methods based on high frequencies guided waves suffer from the limitations of

- The methods are active and require a voltage supply, signal amplifier, function generator, etc., to produce excitation signals within a specific high-frequency range (usually 50–300 kHz).
- The response to high-frequency excitation should be acquired and stored at a high sampling rate to preserve useful information and keep the required resolution of the signal.
- For most high-frequency methods, the delamination should be in the path between the excitation source (actuator) and the location for measurement (sensor).

The first requirement demands for more hardware in the damage assessment technology. The second condition requires a large amount of memory and a high-performance data acquisition system. The third condition demands more sensors and subsequent data acquisition and storage systems to cover all the possible paths between the actuator and sensor for damage-prone areas.

Contrary to high-frequency methods, the proposed method employs structural vibration (0–1000 Hz) obtained through smart elements. The structural vibration is more readily available during the service condition of the structure without the need for any specific range of excitation. Though the attributes of structural vibration (natural frequency, modal damping, FRF, etc.) are global and cannot be employed for the localization of delamination, the proposed approach attempts to extract global and local information through deep learning models. The following sections show details of the general workflow used.

### 2.1. Mathematical Model and Simulated Data

**Figure 2:**
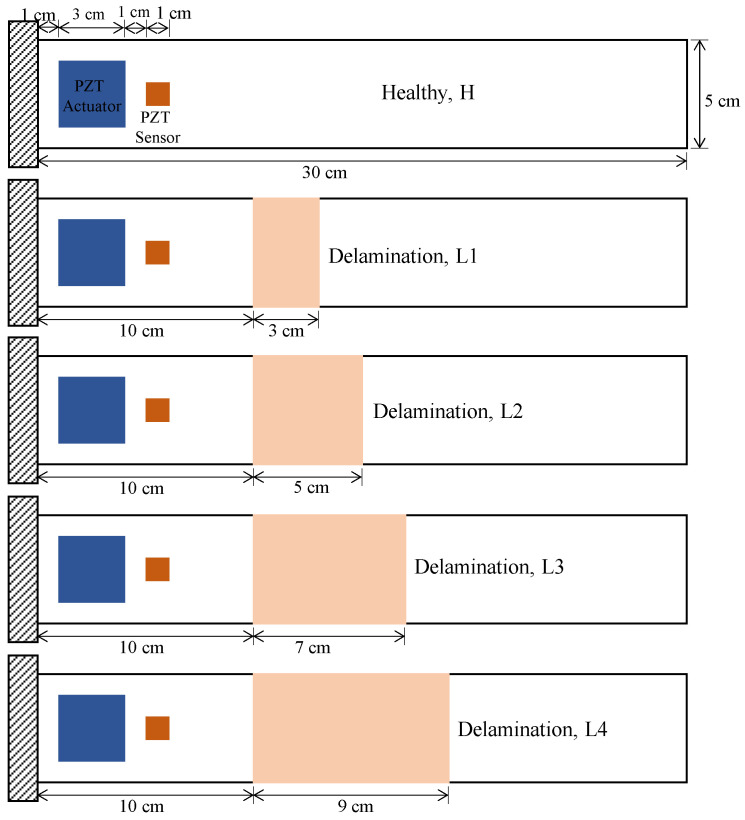
Geometric configuration of laminated composite plate in numerical simulations.

The proposed approach outlined in Figure 1 was carried out using both simulated and experimental data. Improved layerwise theory was employed to simulate delamination in smart composite laminates. A laminated composite plate with 16 plies stacked in a cross-ply symmetric configuration [0/90]_4s_ was modeled in the numerical simulation. A piezoelectric actuator and sensor were modeled on the surface of the laminated plate as shown in Figure 2.

Here, the piezoelectric actuator is attached near the fixed end to excite the plate with low-frequency structural vibrations and the corresponding response is obtained through the piezoelectric sensor. Size of the piezoelectric actuator was 3 × 3 cm^2^ and the dimensions of the piezoelectric sensor were 1 × 1 cm^2^. Four delaminations of different sizes (3 cm–9 cm) were simulated to study the effect the size of the delamination has on the proposed approach. The health states of the composites were denoted by *H* (for healthy), *L*1 (for 3 cm delamination), *L*2 (for 5 cm delamination), *L*3 (for 7 cm delamination), and *L*4 (for 9 cm delamination). An electromechanically coupled finite element model (shown by Equation (1)) of the smart plate was developed using improved layerwise theory [33,34] and high-order electric potential field [35].

(1) $$\left[\begin{array}{cc} M_{uu} & 0\\ 0 & 0 \end{array}\right]\left\{\begin{array}{c} {\ddot{d}}_{u}\\ 0 \end{array}\right\}+\left[\begin{array}{cc} C_{uu} & 0\\ 0 & 0 \end{array}\right]\left\{\begin{array}{c} {\dot{d}}_{u}\\ 0 \end{array}\right\}+\left[\begin{array}{cc} K_{uu} & K_{u\phi }\\ K_{\phi u} & K_{\phi \phi } \end{array}\right]\left\{\begin{array}{c} d_{u}\\ d_{\phi } \end{array}\right\}=\left\{\begin{array}{c} F_{u}\\ F_{\phi } \end{array}\right\}$$

where *d_u_* and *d_ϕ_* denote the displacement and electrical variables at nodal positions, respectively. *M_uu_* is the mass matrix, *C_uu_* is the damping matrix, and *K_uu_* is the stiffness matrix of the system. The electromechanical coupling of the mechanical and electrical field are denoted by *K_uϕ_* and *K_ϕu_*, respectively, while *K_ϕϕ_* is the dielectric stiffness matrix. The terms *F_u_* and *F_ϕ_* denote the respective mechanical force and electric field vectors. A detailed derivation of the finite element model in Equation (1) can be found in references [34,36].

In Equation (1), the electrical excitation, and the corresponding response of the plates in Figure 2 are obtained through the piezoelectric actuator and piezoelectric sensor using the electromechanical coupling matrices, *K_uϕ_* and *K_ϕu_*. These coupling matrices account for the converse and direct piezoelectric effects, where the piezoelectric actuator produces mechanical actuation when a voltage is applied, while the piezoelectric sensor generates a voltage signal when it is mechanically deformed. The matrix form of Eq. 1 can be modified through matrix condensation to obtain the governing equation in the following form.

(2) $$M_{uu}{\ddot{d}}_{u}+C_{uu}{\dot{d}}_{u}+Kd_{u}=F$$

where the modified stiffness matrix *K* and force vector *F* are expressed as follows

(3) $$K=K_{uu}-K_{u\phi }K_{\phi \phi }^{-1}K_{\phi u},F=F_{u}-K_{u\phi }K_{\phi \phi }^{-1}F_{\phi }$$

The thickness of the laminated plate was 0.2 cm and all the delaminations were considered to have occurred in the mid-plane of the laminated composite plate. The material properties of a lamina in the host laminated composite and of the piezoelectric elements used in the numerical simulations are shown in Table 1 and Table 2, respectively.

The numerical model in Equation (2) was implemented using MATLAB by discretizing the piezobonded laminated plate into 60 × 30 elements along the length and width, respectively. Time domain response was obtained from healthy and delaminated smart plates while solving the electromechanically coupled model via Newmark’s time integration algorithm [37]. The plates in each of the five health states (*H*, *L*1, *L*2, *L*3, *L*4) were excited using 10 statistically independent random excitations through the piezoelectric actuator over a period of 2 s, the corresponding responses were obtained through the piezoelectric sensor. The time step chosen for Newmark’s time integration was 0.0001 s in order to maintain numerical accuracy. The data from the simulation was comprised of 50 time series signals, 10 for each of the five health states (10 random excitations × 5 health states × 1 sensor for each health state). The essence of the simulated data is that it contains response from the plates with delaminations of different lengths (from 3 cm to 9 cm).

### 2.2. Experimental Setup and Data

**Figure 3:**
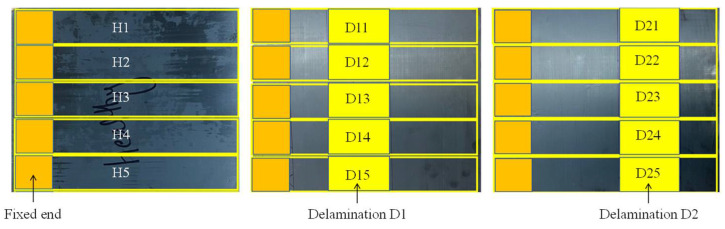
Healthy and delaminated samples representing three health states manufactured using a hot press machine.

To validate the results of the proposed approach experimentally, healthy and delaminated composite samples were manufactured in a hot press machine. Samples with three different states were produced for the experiments: healthy H, delamination D1, and delamination *D2*. The healthy sample had all of its plies perfectly bonded. The delaminated D1 and D2 samples were seeded in their mid-plane with Teflon film (PTFE film (Model KSC-V1000), thickness: 0.03 mm, heat resistance: up to 280 °C) near the free and fixed end of the cantilever beams. The sizes of both delaminations were chosen to be 10 cm in length and 5 cm in width. T700SC-12k-60E carbon fiber prepreg was cut into 35 cm × 30 cm plies and stacked in a symmetric cross-ply configuration of [0/90/0/90]_s_ with a total of 8 plies used in each sample. The properties provided by the manufacturer for the fiber are density 1.8 g/cm^3^, tensile strength 4900 MPa, Tensile Modulus 230 GPa, elongation 2.1 %, thermal conductivity 9.4 W/m.K, filament diameter 7 μm, and epoxy resin. The healthy and delaminated laminated composite plate samples were cured in the hot press machine for 155 min using the curing cycle suggested by the manufacturer. The cured laminated composite plates were cut to a size of 30 cm × 5 cm with a water jet cutting machine to obtain 5 samples for each health state, as shown in Figure 3.

**Figure 4:**
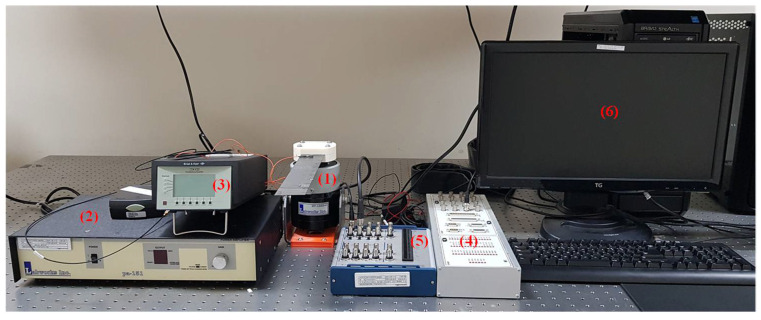
Various apparatus used in the experimental vibration testing; (**1**) shaker; (**2**) amplifier for the shaker; (**3**) amplifier for accelerometer; (**4**) DAQ1; (**5**) DAQ2; (**6**) Lab-view PC.

**Figure 5:**
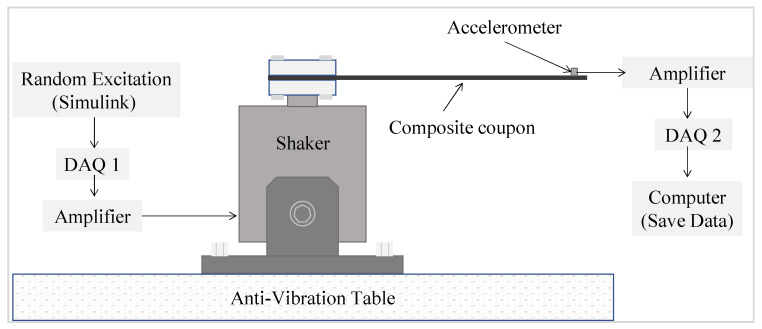
Experimental workflow for vibration testing of composite samples.

Here, the letter H is used to denote a healthy sample while the preceding numbers from 1–5 are used to identify the individual samples. The first digit after the D for the delamination samples D1 and D2 refers to the health state while the second digit identifies the individual samples. Five samples from each health state were used to account for manufacturing and measurement uncertainties. Vibration tests were conducted on the manufactured samples. Figure 4 and Figure 5 show the experimental setup and experimental workflow, respectively, for the random vibration testing.

The experimental setup consisted of three basic systems: excitation, vibration, and response data acquisition systems. The excitation apparatus consisted of a Lab-view PC that generates random signals using MATLAB Simulink. For the excitation of the laminated composite samples, an acquisition system (DAQ 1 (dSPACE/CLP1104)), a signal amplifier (Labworks/PA-151, Labworks Inc., Costa Mesa, CA, USA) for the shaker, and a shaker (Labworks/ET-126-4, Labworks Inc., Costa Mesa, CA, USA) were used to excite the composite beams with the generated random signals.

The laminated composite beams (H, D1, and D2) were clamped using a 3D printed jig to apply cantilevered boundary conditions to the vibrating system. The response acquisition system was used to acquire data from the vibrating coupon. This consisted of various devices; an accelerometer (Bruel & Kjaer/Type 4517-C, Brüel & Kjær, Nærum, Denmark) that was installed on the vibrating composite beams, an amplifier (Bruel & Kjaer/Type 2692-0S2, Brüel & Kjær, Nærum, Denmark), and a data acquisition system (DAQ 2 (NI/USB-6341)). The responses to the random excitations was measured near the clamped end of the healthy and delaminated samples over 15 s. The experimental data reflects the responses caused by delaminations of the same size but in different locations which could be useful evaluating the possibility of determining the location of the delamination through this kind of data.

## 3. Results and Discussion

This section discusses the classification results into different health states for the laminated composite samples in the simulations and experiments based on both handcrafted statistical features and autonomously extracted features.

### 3.1. Classification Results on Handcrafted Statistical Features

Time and frequency domain statistical features of the signals have been used to discriminate between different health states of bearings, gearboxes, and rotating machines [38,39,40,41]. However, statistical features may not allow us to discriminate between the health states of laminated composites where the dynamic response is dominated by the excitation forces and the response signal may not reveal sufficient information to diagnose the characteristics of the fault present when analyzed in a conventional manner. To show the feasibility of using handcrafted statistical features to discriminate between different health states in the simulated data, time and frequency domain features were extracted from the time series data and analyzed through various machine learning algorithms. A window function of 0.2 s was employed to divide the random response signals over the 2 s period into 10 chunks. The purpose of this division is to look for discriminative features in a smaller portion of the signals instead of looking at the entire signal. The handcrafted time domain features used were the mean, standard deviation, skewness, kurtosis, peak to peak values, root mean squared values, crest factor, shape factor, impulse factor, margin factor, and energy of the signal. The extracted frequency domain features were the spectral kurtosis, spectral mean, spectral standard deviation, spectral skewness, and spectral kurtosis. The fifteen features were extracted from numerical data on the composite laminate samples in all five health states. The data from all samples in the various health states amounted to 1000 instances with 15 features for each instance. The values for the hand-crafted features in each instance was processed with different machine learning algorithms, Table 3 depicts the performance of the different classifiers used to process those values in terms of training/validation accuracy and area under the receiver operating characteristics curve (ROC area).

All the classifiers were trained using a 10-fold cross-validation strategy. The results in Table 3 show that the maximum classification accuracy using these statistical features was 43.8% achieved by Fine KNN. However, the ROC area, which reflects the tradeoff between the true positive and false positive rates of the classifiers, indicates that Fine KNN is susceptible to overfitting and will not generalize well to new, unseen data.

**Figure 6:**
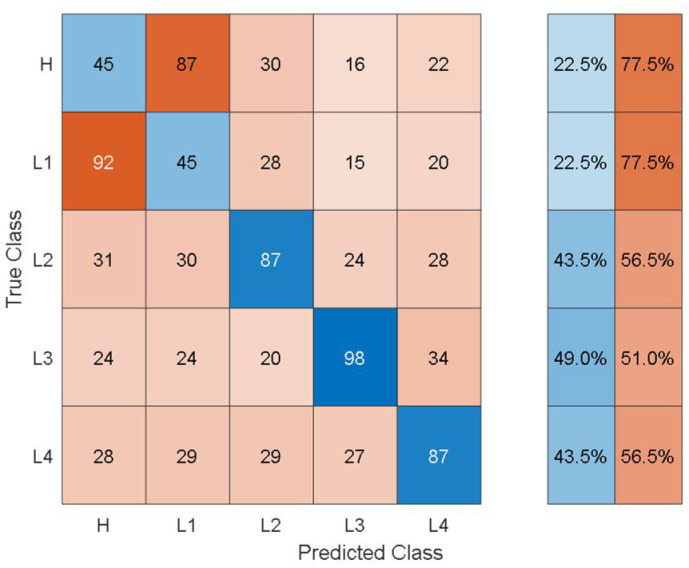
Training/validation confusion matrix of cubic SVM using handcrafted statistical features from simulated data.

Although the classification accuracy is not high enough for practical applications, the training/validation confusion matrix may provide additional insights into the classification performance achieved. Figure 6 shows the training/validation confusion matrix of Cubic SVM.

In the confusion matrices, the values on the main diagonal denote correctly classified instances and the off-diagonal cells contain the misclassified instances. The blue and red columns on the right side show the recall (true-positive rate) and false-negative rate respectively, for each of the health states. Quantitatively, the recall gives the successful detection and isolation of a given health state while the false negative gives the susceptibility of a model to confusing other health states with the actual health state. From Figure 6, it can be observed that though the classification accuracy is not very high, the classification results are consistent with the physics of the problem. For instance, the classifier can distinguish more severe cases of delamination (L2, L3, L4) with relatively higher classification accuracy and the major loss of accuracy comes from confusion between healthy and less severe cases of delamination i.e., confusion between H and L1.

Experimental data was collected over a period of 15 s, the signal for each sample in each health state was split into 20 chunks using a window of 1875 data points. This created 100 instances for the five samples in each health state, resulting in a total of 300 instances for the three health states. The same statistical features used with the simulated data were extracted from the experimental data and were again processed using various machine learning algorithms. Table 4 shows the performance results for each of the classifiers in terms of training/validation accuracy and area under the receiver operating characteristics curve (ROC area). All classifiers were trained using a 10-fold cross-validation strategy.

The result in the table shows that delaminations of the same size but at different locations can be correctly classified with a maximum classification accuracy of around 70 %. However, a classifier trained with this accuracy may be susceptible to high misclassification rates when deployed for making predictions on new data.

**Figure 7:**
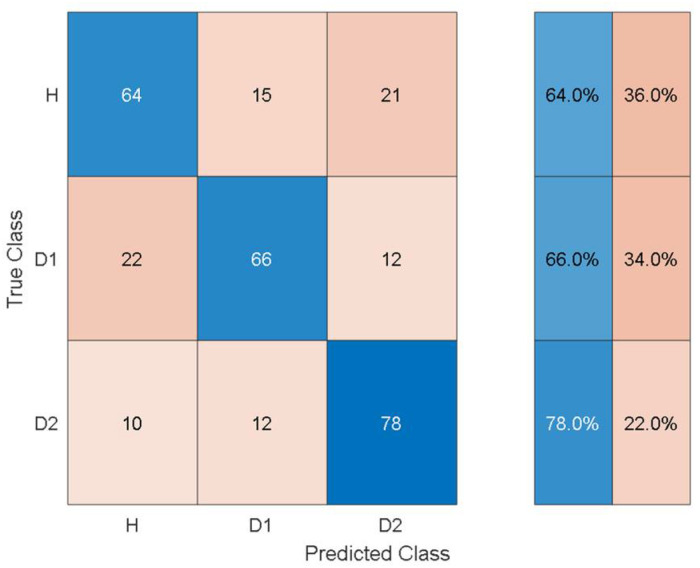
Training/validation confusion matrix for the Fine Tree classifier using handcrafted statistical features from experimental data.

To give an idea of the per-class misclassification results, Figure 7 depicts the training/validation confusion matrix for the Fine Tree classifier.

It can be observed that the classifier can distinguish the three health states with 64%, 66%, and 78% classification accuracy. Furthermore, the susceptibility to misclassification was 36%, 34%, and 22% for the three health states.

Although the statistical features provide physically consistent results, the low classification accuracy when using these features hinders their practical application for the detection and isolation of delamination from low-frequency structural vibration in laminated composites. The next section shows the results of using discriminative features that were extracted autonomously via pretrained deep learning models.

### 3.2. Results from Using Features Extracted Autonomously Using Pretrained Deep Learning Models

In the deep learning framework, pretrained models can be employed for three purposes: to make predictions on new unseen data that is similar to previous data, for feature extraction using the activations of deep layers as features, and for transfer learning based on fine tuning a network that was pretrained on data from a different but related task to work with limited new data [42,43]. In this work, pretrained deep learning models based on AlexNet, GoogleNet, SqueezeNet, ResNet-18, and VGG-16 were employed for autonomous feature extraction from the limited data gathered by simulations and experiments. The reason for choosing different pretrained models for the current problem was to show the effect of the architecture, depth, and the number of pretrained deep learning model parameters for the fault diagnosis in a transfer learning framework. The characteristics features of the adopted pretrained models are shown in Table 5.

Herein, it is observed that each network has different architectural characteristics, and one can choose a pretrained model based on the initial assessment of the results from different models, depth of the models, memory size, predictive performance, and prediction speed. A detailed description of the architecture of different pretrained deep learning models can be found in the references [44,45,46].

The mathematical details of autonomous feature extraction via pretrained deep learning models are not discussed here for the sake of brevity but can be found in references [47,48,49]. The autonomous features were processed via a quadratic support vector machine using 10-fold cross validation and a one-vs-all training strategy for the detection, quantification, and localization of delamination in laminated composites. The mathematical details of SVM for the assessment of discriminative features and classification results can be found in the references [50,51,52].

**Figure 8:**
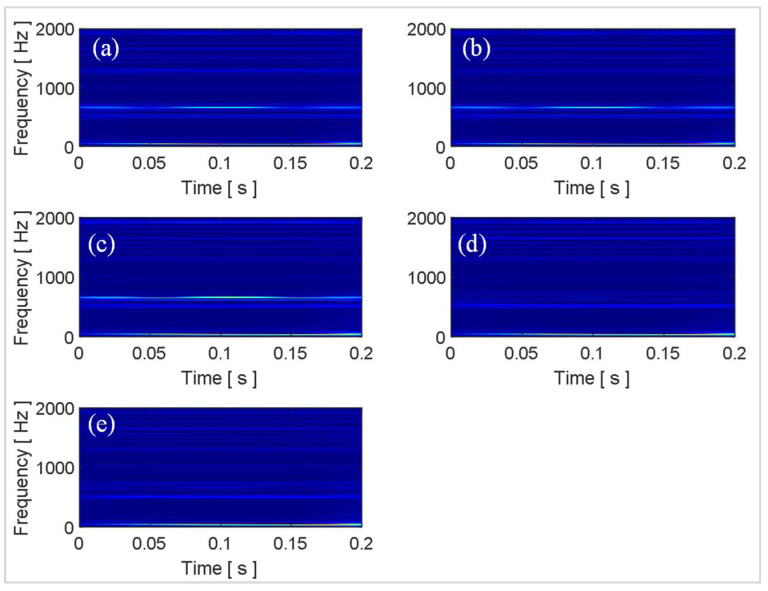
Some representative time-frequency images of the simulated data obtained via the SET technique: (**a**) H; (**b**) L1; (**c**) L2; (**d**) L3; (**e**) L4.

Since the existing deep learning models were pretrained on image data, the time domain signals were transformed into time-frequency images using the Synchroextracting Transform (SET) technique introduced by Yu et al. [26]. The reason for choosing SET instead of short-time Fast Fourier Transforms (STFT) or wavelet scalograms was that SET provided better time-frequency resolution. The mathematical details of encoding time series data into an image with SET can be found in references [26,27]. The SET window length is the parameter that affects the time-frequency resolution. In the current work, after trying various lengths of SET windows, a window length equal to the length of the signal was found to provide the best time-frequency resolution for laminated composites. The time series data, windowed into smaller chunks in the same way as for the statistical features, was processed with SET to obtain time-frequency images representing the simulated and experimental data. Some representative time-frequency images of the simulated data obtain through SET are shown in Figure 8.

The time-frequency images show different characteristic spectrums for each of the five health states. However, due to the random nature of the response signals, it is difficult to differentiate the health states from their time-frequency images using the naked eye. Moreover, the images in Figure 8 are some representative examples from the 100 images for each health state and only correspond to a single random response signal. The difficulty level is further increased as the number of random excitations and their corresponding responses increase. Owing to their inherent architecture, deep learning models can look for minute differences between images allowing them to differentiate similar looking images autonomously with high accuracy. In the current work, the total number of images is only 500 (100 images for each health state), which is not sufficient to optimize the parameters of a deep learning model which is being developed and trained from scratch. Hence, in this work, off-the-shelf pretrained deep learning models were employed for autonomous feature extraction.

The autonomous features extracted at different layers of the pretrained models have different dimensions and discriminative capabilities. In the current work, features from different layers of the pretrained models are leveraged to discriminate between different health states of the laminated composites based on the simulated and experimental data. To ensure a consistent parametric study, the autonomous features were processed with quadratic SVM, Table 6, Table 7, Table 8, Table 9 and Table 10 show its performance based on the autonomous features extracted from the simulated data in terms of training/validation accuracy, test accuracy, and the number of autonomous features.

**Figure 9:**
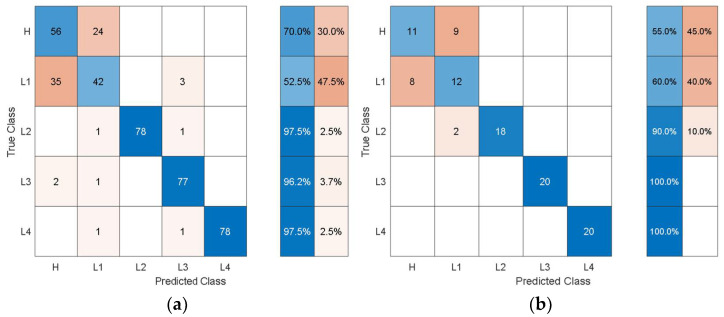
Classification performance of SVM using the autonomous features from the 23rd layer of AlexNet in terms of its confusion matrices: (**a**) training; (**b**) test.

**Figure 10:**
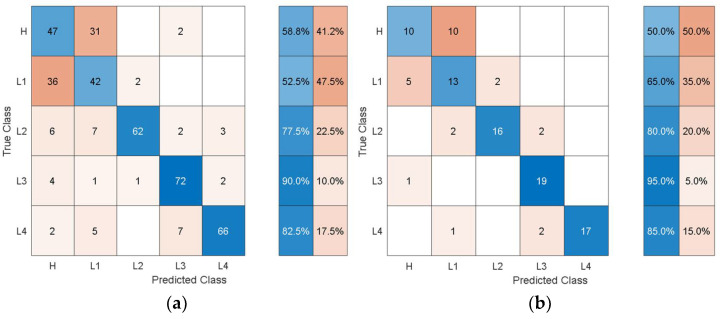
Classification performance of SVM using the autonomous features from the 142nd layer of GoogleNet in terms of its confusion matrices: (**a**) training; (**b**) test.

**Figure 11:**
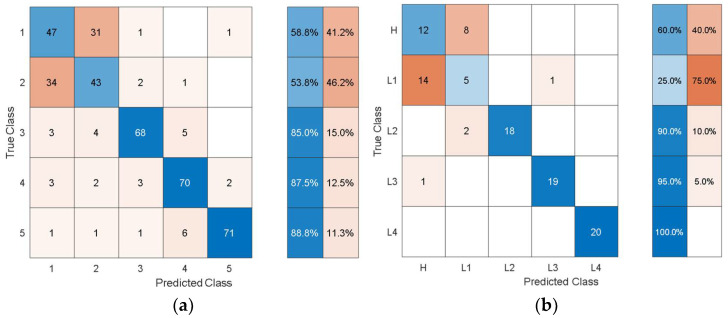
Classification performance of SVM using the autonomous features from the 66th layer of SqueezeNet in terms of its confusion matrices: (**a**) training; (**b**) test.

**Figure 12:**
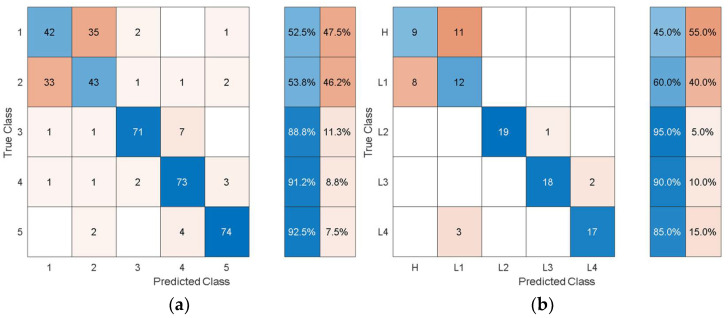
Classification performance of SVM using the autonomous features form the 68th layer of ResNet-18 in terms its confusion matrices: (**a**) training; (**b**) test.

**Figure 13:**
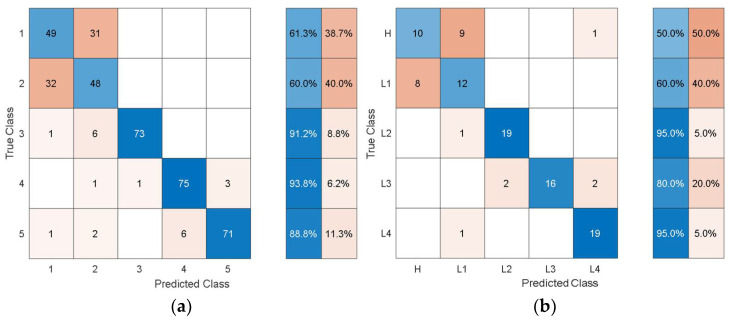
Classification performance of SVM using the autonomous features from the 39th layer of VGG-16 in terms of its confusion matrices: (**a**) training; (**b**) test.

It can be observed that the dimensions of the discriminative features are increasing as the feature extraction layer is moved from the final layers to the inner layers towards the initial layers of the pretrained models. In this problem, the accuracy is not affected much by whether high-level features (i.e., features from the last layers) or low-level features (i.e., features from the inner and initial layers) are used. However, the dimensions and consequently the computational cost of using the low-level features are higher than for using the high-level features. When compared with the classification performance using the hand-crafted statistical features shown in Table 3, the performance using the autonomous features has substantially increased for all the pretrained models. To get further insights into the classification performance, Figure 9, Figure 10, Figure 11, Figure 12 and Figure 13 depict the confusion matrices for the SVMs with the highest accuracy seen in Table 6, Table 7, Table 8, Table 9 and Table 10 coming from using the autonomous features of the pretrained models.

The dataset from the simulation was split into 80% training data and 20% test data. In the confusion matrices, the values on the main diagonals denote correctly classified instances and the off-diagonal cells contain misclassified instances. The blue and red columns on the right depict the recall (true-positive rate) and false-negative rate, respectively, of the actual classes. Quantitatively, the recall gives the successful detection and isolation of a given health state while the false negative gives the susceptibility of a model to confusing other health states with the actual health state. Specifically, from Figure 9, the autonomous features extracted by Alexnet can distinguish healthy cases H from all other health states with 70% accuracy, but it is susceptible to incorrectly classifying other health states as H at a rate of 30%. The values on the main diagonal and in the off-diagonal cells show that the model is susceptible to the incorrect classification of L1 as H.

From all the confusion matrices, it can be observed that the per-class training performance has substantially increased when using the autonomously extracted features from each of the pretrained models when compared with the training performance using the handcrafted statistical features, as shown in Figure 6. Moreover, for all the pretrained models, the major losses of accuracy are associated with confusion between the healthy case H and the least severe case of delamination L1. The physical reason for the confusion of H with L1 is that the 3 cm delamination is only causing a very subtle change in the structural stiffness and consequently in the structural dynamic response when compared with the healthy case. The close resemblance in the characteristic dynamic responses of L1 and H causes the classifier to confuse the two cases. Moreover, from the test confusion matrices, it can be observed that the correct prediction rate from using the pretrained model to autonomously extract features, regardless of the model used, is reasonably high and show similar behavior to that shown by the training confusion matrices. The results for the increasing size of delamination and the ease in its detectability in the current work are supported by the research finding of the articles in the references [50,53].

For the experimental data, the structural vibration data windowed into the same smaller chunks used to extract the statistical features from was encoded to time-frequency images using SET. The image data based on the experimental results was processed using pretrained deep learning models to autonomously extract features. The features were extracted through the last layers of the models and were processed with quadratic SVM using 10-fold cross-validation and a one-vs-all training strategy. The experimental data was randomly split into 80% training and 20% test data. The classification results from the SVM using autonomous features are shown in Table 11.

**Figure 14:**
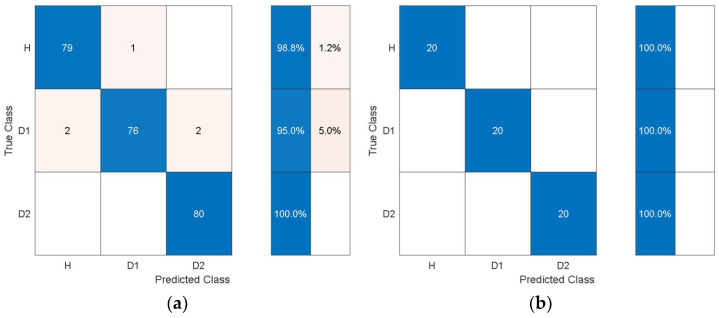
Classification performance of SVM using the autonomous features form the 23rd layer of AlexNet in terms of its confusion matrices: (**a**) training; (**b**) test.

**Figure 15:**
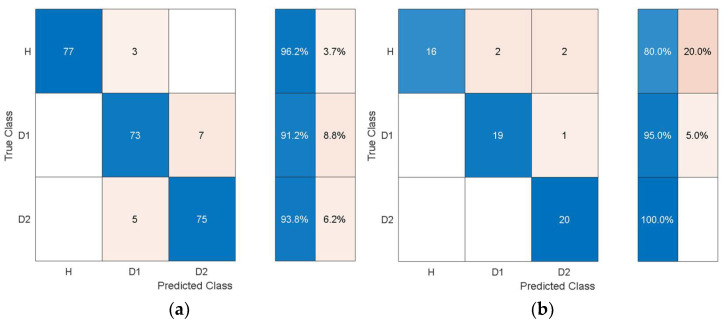
Classification performance of SVM using the autonomous features from the 142nd layer of GoogleNet in terms of its confusion matrices: (**a**) training; (**b**) test.

Comparing Table 11 with Table 4 shows that the overall training accuracy has substantially increased when using the autonomous features from all the pretrained models. Among the autonomous features extracted by the various pretrained models, the features extracted by AlexNet achieve the best classification performance, while the worst classification performance was observed when using the features from GoogleNet. For a detailed look at the classification performance using features from AlexNet and GoogleNet, Figure 14 and Figure 15 show the training and test confusion matrices for the SVM trained on the features extracted by AlexNet and GoogleNet, respectively.

It can be observed that the SVM can distinguish healthy cases from delaminated cases with 98.8% accuracy while it is only susceptible to incorrectly classify other health states as healthy at a rate of 1.2%. The minimum per-class classification accuracy was 95.0% for D1 delamination samples using the features from AlexNet while the maximum per-class classification accuracy was 100% for D2 delamination samples. For the pretrained model with the worst overall classification performance (GoogleNet), the minimum per-class accuracy was 91.2% for D1 delamination samples and the maximum per-class accuracy was 96.2% for the healthy H samples. In general, the autonomous features from all the pretrained models can be used to distinguish between delaminations of the same size occurring at different locations while they can also be used to distinguish healthy cases H from delaminated cases D1 and D2 with higher accuracy. The experimental data has accounted for the uncertainty in the manufacturing of coupons and measurement error by considering five samples of each health state. The existing literature mostly focuses on modal parameters (natural frequencies, modes shapes, mode shape curvature) of structural vibration to assess delamination [54]. However, modal parameters are global, and the experimental measurement of mode shapes is a difficult task.

The current work showed the feasibility of limited structural vibration data for the detection, localization, and quantification of delamination through classification in a supervised learning framework. The proposed approach could be employed to assess the size and location of delamination by predicting a label for a given health state which can be interpreted for the size and location of a new delamination. For instance, the label for a delamination of size 6 cm would be either L2 (delamination of 5 cm) or L3 (delamination of 7 cm) due to their similar response characteristics. In general, the delamination of different sizes near the free and clamped ends may have the same dynamics response characteristics, and the new delamination may be entirely different from the one considered in the pretrained model. Moreover, the labels for the prospective delamination may not be known. The future extension of the current work will adopt a more generic approach by using more than one sensor along the length of the specimen and an unsupervised framework for the detection, localization, and quantification of delamination. These results show our approach could be beneficial for delamination localization based on low-frequency structural vibration data.

## 4. Conclusions

This study investigated the effectiveness of autonomous discriminative features via pretrained deep learning models for assessing delamination in laminated composites. The simulations and experiments were carried out to obtain low-frequency random structural vibration data from the sample in either healthy or delaminated states. The vibration data was encoded into high-resolution time-frequency images using the Synchroextracting Transform (SET) technique. This image data was then processed with various pretrained deep learning models (AlexNet, GoogleNet, SqueezeNet, ResNet-18, 491 VGG-16) for autonomous discriminative features, and a support vector machine (SVM) was employed to assess the health state based on those features. The autonomous features were found to outperform the handcrafted statistical features for diagnosing delamination in laminated composites. The work also compared the feasibility of low- and high-level features of pretrained models for delamination assessment. The high-level features were found to perform relatively better than the low-level features. The proposed approach eliminates the requirement of extensive training data and labor-intensive process of human-engineered statistical features. The method has the potential for the autonomous diagnosis and prognosis of defects in modern composite structures.

## Figures and Tables

**Table 1:** Material properties of a lamina in the host laminate.

E_1_	E_2_, E_3_	G_12_, G_13_	G_23_	ρ	ν_12_, ν_13_	ν_23_
372 GPa	4.12 GPa	3.99 GPa	3.6 GPa	1788.5 kg/m^3^	0.275	0.42

**Table 2:** Material properties of the piezoelectric elements.

E	ν	ρ	d_31_, d_32_	d_24_, d_15_	b_11_
62 GPa	0.30	7500 kg/m^3^	274 × 10^−12^ m/V	−741 × 10^−12^ m/V	14.41 nf/m

**Table 3:** Classification performance of different algorithms on data from simulation using handcrafted statistical features.

Classifier	Training/Validation Accuracy %	ROC Area
Fine Tree	36.1	0.58
Linear Discriminant	21.7	0.50
Kernel Naïve Bayes	26.4	0.53
Cubic SVM	36.2	0.51
Fine KNN	43.8	0.47

**Table 4:** Classification performance of the various algorithms using handcrafted statistical features from experimental data.

Classifier	Training/Validation Accuracy %	ROC Area
Fine Tree	69.3	0.78
Linear Discriminant	63.0	0.65
Kernel Naïve Bayes	66.7	0.70
Cubic SVM	61.7	0.72
Fine KNN	57.0	0.60

**Table 5:** Comparison of pretrained deep learning models.

Network	Number of Layers	Parameters (Millions)	Memory Size (MB)	Architectural Building Blocks of Hidden Layers
AlexNet	25	61.0	227	Convolution, ReLU, Pooling, Cross Channel Normalization, Grouped Convolution, Dropout, Fully Connected Layers, series connections
GoogleNet	144	7.0	27	Convolution, ReLU, Pooling, Cross Channel Normalization, Inception modules, multi-scale convolutional transformations, convolution layers replaced with small blocks
SqueezeNet	68	1.24	5.2	Convolution, ReLU, Pooling, Depth Concatenation (with squeeze and expand layers), Simple and Complex Bypass
ResNet-18	71	11.7	44	Convolutions, Batch Normalization, ReLU, Pooling, Addition, Residual blocks as structural unit
VGG-16	41	138	515	Convolution, ReLU, Pooling, Drop out, fully connected layers, smaller filters, and series connections

**Table 6:** Classification performance of SVM using the autonomous features from AlexNet.

**AlexNet**	**Layer Number**	**Name of Feature Extraction Layer**	**Training/Validation Accuracy %**	**Test Accuracy %**	**Number of Features**
	23	‘fc8’	77.25	75.0	1000
	19	‘fc6’	82.75	81.0	4096
	9	‘pool2’	72.25	77.0	43,364
	5	‘pool1’	70.0	73.0	69,984

**Table 7:** Classification performance of SVM using the autonomous features from GoogleNet.

**GoogleNet**	**Layer Number**	**Name of Feature Extraction Layer**	**Training/Validation Accuracy %**	**Test Accuracy %**	**Number of Features**
	142	‘loss3-classifier’	72.25	75.0	1000
	136	‘inception_5b-pool’	64.75	69.0	40,768
	122	‘inception_5a-pool’	66.50	77.0	40,768
	93	‘inception_4d-pool’	67.25	66.0	100,352

**Table 8:** Classification performance of SVM using the autonomous features from SqueezeNet.

**SqueezeNet**	**Layer Number**	**Name of Feature Extraction Layer**	**Training/Validation Accuracy %**	**Test Accuracy %**	**Number of Features**
	66	‘pool10’	74.75	74.0	1000
	53	‘fire8-expand3x3’	70.25	74.0	50,176
	34	‘pool5’	70.75	77.0	50,176
	19	‘pool3’	71.25	72.0	100,352

**Table 9:** Classification performance of SVM using the autonomous features from ResNet-18.

**ResNet-18**	**Layer Number**	**Name of Feature Extraction Layer**	**Training/Validation Accuracy %**	**Test Accuracy %**	**Number of Features**
	68	‘pool5’	75.75	75.0	512
	59	‘res5a_branch1’	72.25	73.0	25088
	45	‘res4b_branch2a’	71.75	71.0	50176
	13	‘res2b_branch2a’	75.0	79.0	200704

**Table 10:** Classification performance of SVM using the autonomous features from VGG-16.

**VGG-16**	**Layer Number**	**Name of Feature Extraction Layer**	**Training/Validation Accuracy %**	**Test Accuracy %**	**Number of Features**
	39	‘fc8’	77.25	78.0	1000
	33	‘fc6’	79.0	76.0	4096
	25	‘pool4’	70.25	77.0	100,352
	11	‘pool2’	76.50	76.0	401,408

**Table 11:** Classification performance of SVM using the autonomous features from various pretrained models.

Pretrained Model	Feature Extraction Layer	Training/Validation Accuracy %	Test Accuracy %	Number of Features
AlexNet	‘fc8’	97.92	100	1000
GoogleNet	‘loss3-classifier’	93.75	91.67	1000
SqueezeNet	‘pool10’	96.25	98.33	1000
ResNet-18	‘pool5’	95.83	98.33	512
VGG-16	‘fc8’	97.08	98.33	1000

## Data Availability

The data that support the findings of this study is available from the corresponding author upon request.
